# Methodologyfor Molecular Dynamics Simulation of Plastic Deformation of a Nickel/Graphene Composite

**DOI:** 10.3390/ma15114038

**Published:** 2022-06-06

**Authors:** Karina A. Krylova, Liliya R. Safina, Stepan A. Shcherbinin, Julia A. Baimova

**Affiliations:** 1 Institute for Metals Superplasticity Problems of the Russian Academy of Sciences, Khalturina 39, Ufa 450001, Russia; saflia@mail.ru (L.R.S.); julia.a.baimova@gmail.com (J.A.B.); 2 Ufa State Petroleum Technological University, Kosmonavtov Str. 1, Ufa 450062, Russia; 3 Peter the Great St. Petersburg Polytechnic University, Polytechnicheskaya Str. 29, St. Petersburg 195251, Russia; stefanshcherbinin@gmail.com; 4 Bashkir State University, Validy Str. 32, Ufa 450076, Russia

**Keywords:** crumpled graphene, nickel/graphene composite, molecular dynamics, mechanical properties

## Abstract

In this study, some features of molecular dynamics simulation for evaluating the mechanical properties of a Ni/graphene composite and analyzing the effect of incremental and dynamic tensile loading on its deformation are discussed. A new structural type of the composites is considered: graphene network (matrix) with metal nanoparticles inside. Two important factors affecting the process of uniaxial tension are studied: tension strain rate (5 $\times \;10^{-3}$ ps${}^{-1}$ and 5 $\times \;10^{-4}$ ps${}^{-1}$) and simulation temperature (0 and 300 K). The results show that the strain rate affects the ultimate tensile strength under tension: the lower the strain rate, the lower the critical values of strain. Tension at room temperature results in lower ultimate tensile strength in comparison with simulation at a temperature close to 0 K, at which ultimate tensile strength is closer to theoretical strength. Both simulation techniques (dynamic and incremental) can be effectively used for such a study and result in almost similar behavior. Fabrication technique plays a key role in the formation of the composite with low anisotropy. In the present work, uniaxial tension along three directions shows a big difference in the composite strength. It is shown that the ultimate tensile strength of the Ni/graphene composite is close to that of pure crumpled graphene, while the ductility of crumpled graphene with metal nanoparticles inside is two times higher. The obtained results shed the light on the simulation methodology which should be used for the study of the deformation behavior of carbon/metal nanostructures.

## 1. Introduction

The remarkable properties of crumpled graphene make it an ideal candidate for high-performance structures [1,2,3] since it has a high specific surface area, porosity, mechanical properties, etc. [4,5]. Recently, crumpled graphene (CG) has attracted much attention as a potential material to fabricate a composite with different metal nanoparticles due to its unique properties [6]. For example, metals such as Ni, Au, and Pt are used in graphene fabrication as catalysts and, thus, can be precursors for new composite carbon structures [6,7,8]. Nickel has been both experimentally and numerically analyzed as a promising metal for the fabrication of composites with improved strength and ductility [9,10,11]. It is shown that even a small amount of graphene added to the metal matrix can significantly improve its mechanical properties [12,13,14].

Improvement of the strength of metals is of high importance for the development of structural materials and carbon polymorphs are shown to be a prospective candidates for such enhancement [15]. The first attempts to integrate graphene into metal matrix composites showed that size, number of layers, and type of settlement of graphene in the metal matrix can considerably affect the mechanical strength of the resulting composite [13,16,17]. For example, the hardness (maximum elastic strain) of the composite decrease (increases) with an increase in the number of graphene layers, which indicates that the mechanical properties of nickel–graphene nanocomposites can be engineered [16]. Different external treatment for improvement of the composite properties was studied by both experimental and simulation techniques like shock treatment [17] and nanoindentation [13,18]. However, metal matrix composites are very well known to date, and new attempts have arisen to study metal/graphene composites with new architecture: on the basement of graphene aerogels, graphene oxide, or crumpled graphene in combination with metal nanoparticles [1,6,9,19]. Such composite materials can have a wide application, for example as corrosion-resistant materials [20], in automobile and aerospace engineering due to high strength [21,22] and wear resistance [23], to name a few.

It was shown that several important factors for superior mechanical properties of metal/graphene composites can be taken into account. For example, if the graphene flakes are agglomerates, it can decrease the mechanical characteristics of the composite [24,25,26]. The other main factor is anisotropy of the structure or the directions of orientation of graphene in the composite. Homogeneous dispersion of graphene inside the metallic matrix is one of the important issues since it is difficult to disperse graphene uniformly into a metal matrix because it tends to agglomerate during processing [26]. A metal/graphene interface can affect the mechanical properties positively or negatively depending on its coherency [24,25,27]. The fabrication technique also affects the mechanical behavior. It was demonstrated that the lateral size and orientation of graphene in the composite strongly affect the deformation mechanisms and their strength [28,29]. If all of these parameters are controlled, metal/graphene composites with superior mechanical properties can be produced [14].

Molecular dynamics (MD) simulation is one of the most widely-used numerical tools in the studies of physical and mechanical properties of nanomaterials since it provides information not readily accessible experimentally. A number of different processes have been explored in carbon-metal systems by MD, including deformation and fracture [30,31,32,33,34], structural transformations [35,36], defects [37] etc. The great advantage of MD simulations is the possibility to check the conditions that cannot be replicated in a real experimental environment. For example, Ref. [38] studied the state-of-the-art understanding of mechanisms controlling the growth and synthesis of carbon nanotubes and graphene using catalytic chemical vapor deposition by MD together with the presentation of the new model for 2D nucleation of a graphene sheet from amorphous carbon on a nickel surface. Moreover, MD calculations can be used to determine the effects of one system variable at a time. For example, the formation of the Ni/graphene composites during sintering was analyzed in detail in Ref. [9], which allows for understanding of the structural transformations in such composites.

Different methods can be used to apply active deformation to the investigated atomic system. One of the key points is to ensure that the applied deformation scheme follows the basic concepts of continuum mechanics such as energy conservation. The chosen deformation scheme can affect the final results, especially when tension and fracture of the sample are considered, and the search for a better deformation technique is the motivation of this paper.

In the present work, the methodology for MD simulation of plastic deformation of a Ni/graphene composite is presented. Two deformation schemes (incremental and dynamic tension) and two very important parameters (strain rate and simulation temperature) are taken into account.

## 2. Materials and Methods

### 2.1. Formation of Ni/Graphene Composite

**Figure 1:**
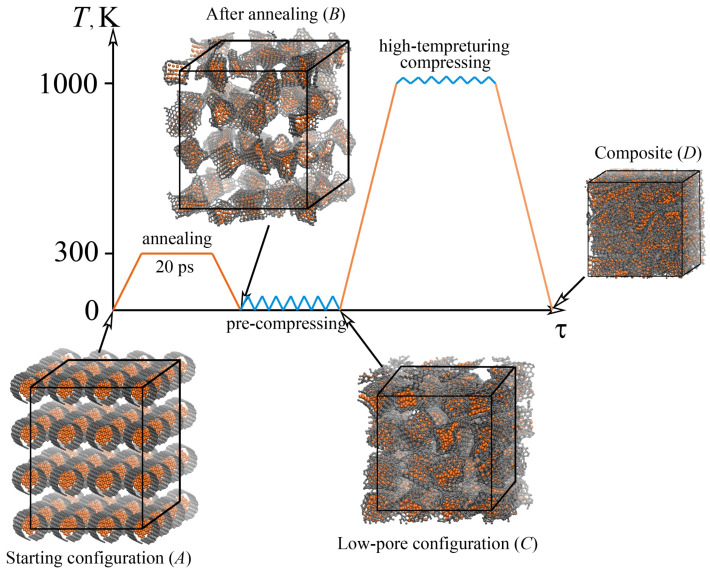
Scheme of the performed tests and their corresponding conditions to obtain a Ni/graphene composite. Snapshots of the structures in the initial (*A*), final (*D*) states, and states after each processing stage (stage *B*, after annealing at 300 K for 20 ps; stage *C*, after pre-compression). Nickel atoms are shown by orange and Carbon atoms, - by grey color.

In the present work, the CG/Ni composite system comprises 19,136 atoms, including 2 parts: the carbon matrix and metal nanoparticle fillers, which are brought together, as shown in Figure 1. The matrix of the composite is composed of a mixture of graphene flakes of the same size (16,128 carbon atoms in total) and Ni nanoparticles inside the cavities of the graphene flakes (3008 Ni atoms in total).

The starting configuration, which is not yet a composite, but just the precursor—a system of graphene flakes with Ni nanoparticles inside each flake—is shown in Figure 1, stage *A*. Note that in previous studies [39,40,41] it was shown that to obtain a Ni/graphene composite with high strength, it is necessary to take into account the difference in the diameter of Ni nanoparticle and the diameter of the rolled graphene flake. If the diameter of the nickel nanoparticle is large, then the graphene flake completely covers the nanoparticle, which makes the formation of strong bonds between the graphene flakes difficult, and therefore, complicates the composite fabrication. Therefore, the size of nickel nanoparticles is one of the key factors. For this study, each particle contains 47 Ni atoms and originally has a round shape with a perfect crystal lattice. A nanoparticle is immersed inside the graphene flake with the help of a homemade program. It should be noted, that for this size of nanoparticle, molecular forces between the nanoparticle and graphene flake prevent bond breaking in the basal plane of flakes. To generate a representative model of the graphene network, 64 graphene flakes are randomly packed into a 3D simulation cell forming the system with randomly oriented structural elements. As it can be seen from Figure 1, stage *A*, the precursor structure is not totally random, and very ideal with round nanoparticles and perfectly rolled graphene flakes, which is far from realistic crumpled graphene. However, the further deformation-temperature treatment allows for finding much more real configurations. The dimensions along *x*, *y*, and *z* directions are 100 Å, 101 Å, and 85 Å, respectively. A more detailed description of the formation of the starting configuration of the Ni/graphene composite is presented in [40,41,42,43].

After creating the initial configuration, three different treatment routes are applied, as shown in Figure 1. During the first route, the samples are heated to room temperature (300 K) and exposed for 20 ps. An increase in the time of annealing by 10 times does not lead to significant structural changes. This step allows obtaining a more realistic structure of the Ni/graphene composite due to the crumpling of graphene flakes and their anisotropic distribution over the simulation cell during annealing. Then the sample is further cooled to zero temperature. The structure of Ni/graphene composite after preliminary annealing is shown in Figure 1, stage *B*. At first (Figure 1, stage *A*), flakes are rotated for the same angle, but after annealing GFs are crumpled in a different way, structural elements (flake with nanoparticle inside) have different shapes and sizes, and the distance between different GFs was changed during temperature treatment as well as the pore size in the structure. This process should provide the fabrication of the composite with low anisotropy. Moreover, it is known that at temperatures lower than 600 K, covalent bonds in the basal plane of graphene are very strong. Thus, even during annealing, only edge atoms of graphene flakes can interact with the edge atoms of neighboring graphene flakes. During energy minimization or annealing, only changes in the shape of graphene flakes can be observed as well as changes in the equilibrium distance between neighboring flakes.

After exposure of the sample at 300 K, an approximation to quasistatic compressive loading in each deformation increment is conducted in two steps, including (i) compression during 27 ps to eliminate the large pores in the structure and (ii) compression to the composite state. The time of compression depends on the porosity of crumpled graphene: pre-compression can be finished when neighboring GFs start to interact and stress in the system becomes non-zero. Compression to the composite state took place before maximum density is achieved in the system. The time of compression depends on the structural peculiarities. The compression rate in the loading process is 5 $\times \;10^{-3}$ ps${}^{-1}$. The loading is applied along all three dimensions simultaneously (i.e., the sample is hydrostatically compressed). After pre-compression, the size of the composite in all three directions (*x*, *y*, and *z*) is the same and equal to 61 Å. At this stage, the deformation of the structure occurs due to a decrease of the free volume (pores and voids) between the structural elements and also a slight compression of graphene flakes and Ni nanoparticles (see Figure 1, stage *C*). However, nickel nanoparticles do not lose their crystal structure during the pre-compression process and remain in the internal cavities of graphene flakes. During pre-compression, the sample was compressed to about 40% in the *x* and *y* directions, and to 28 % in the *z* direction in comparison with the starting configuration.

The second step of deformation—high-temperature compression–is applied at 1000 K to obtain the final composite state (see Figure 1, stage *D*). This temperature is enough to facilitate the formation of new valent bonds between graphene flakes, but not enough to melt Ni nanoparticles [40,41]. The compression rate in the loading process is 5 $\times \;10^{-3}$ ps${}^{-1}$. As a result of high-temperature hydrostatic compression, the composite is deformed for 13% more in comparison to the pre-compressed structure. The resulting deformation corresponds to the maximum possible density of the Ni/graphene composite ($\rho_{c}$ = 6.54 g cm${}^{-3}$), which is almost twice as high as the diamond density ($\rho_{d}$ = 3.55 g cm${}^{-3}$).

After high-temperature compression, the structure is relaxed to minimize the internal elastic stresses that appear during the preparation of the Ni/graphene composite. It should be noted that in early works [40,41,42,43], the relaxation of the structure after the production of the composite is not carried out, which led to the appearance of excess stress, which affects the final mechanical properties of the composite.

### 2.2. Two Approaches to the Tensile Load

To apply the load to the system, boundaries of the simulation cell are moved with the given strain rate, which is the displacement-control method. In this work, two types of a tensile load of a Ni/graphene composite are considered: dynamic and incremental uniaxial tension [44]. For the dynamic types of loading, strain monotonically increases with time, and borders of the simulation cell are displaced continuously. For the incremental type of loading, each step of tension is followed by a step of relaxation during which boundaries of the simulation cell are fixed (to relax stresses without tensile loading).

**Figure 2:**
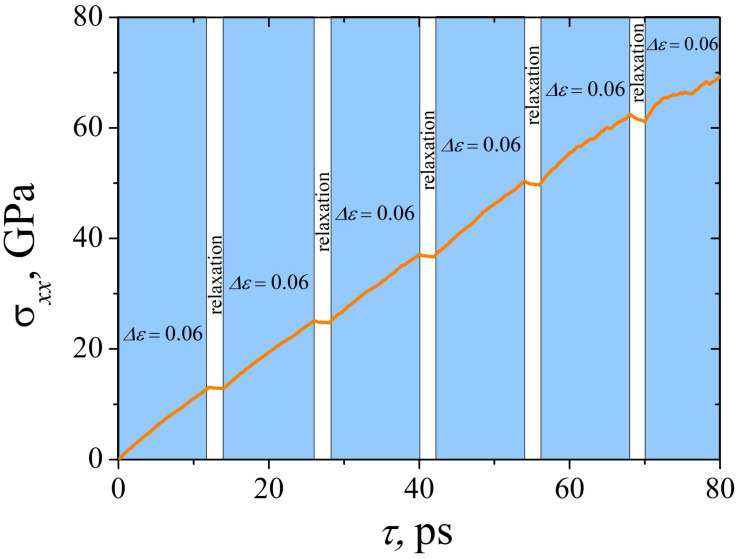
Stress as the function of time for a Ni/graphene composite under uniaxial incremental tension at 0 K and a strain rate of 5 $\times \;10^{-3}$ ps${}^{-1}$. The term “relaxation” means exposure of the structure conserving the volume of the simulation cell for 2 ps (colored white). The areas, when uniaxial tension is applied, are colored blue.

Figure 2 shows the schematic of the incremental loading: uniaxial tensile stress σ as the function of time, where the steps of uniaxial tension are colored blue, and the steps of relaxation are colored white. After each tension step (strain time Δτ, strain value Δε), the Ni/graphene composite is equilibrated for 2 ps, while the external pressure is not decreased to zero, but remains at the same level as during deformation. At the “relaxation” step, the system is equilibrated through the breaking of some bonds between carbon atoms and the formation of new ones. This leads to the redistribution of internal stresses that appeared during tension and even to some stress decrease (see Figure 2).

The strain-controlled uniaxial tension in the *x*-direction is performed to apply two deformation mechanisms. A constant engineering strain rate of two values, 5 $\times \;10^{-3}$ ps${}^{-1}$ and 5 $\times \;10^{-4}$ ps${}^{-1}$, is applied for all the simulations for comparison. Note that, under incremental tension, a decrease in the strain rate by a factor of *n* leads to the same increase in the time of deformation of the composite. For example, the incremental deformation time Δ τ is 12 and 120 ps at strain rates of 5 $\times \;10^{-3}$ ps${}^{-1}$ and 5 $\times \;10^{-4}$ ps${}^{-1}$, respectively. However, the time of the “relaxation” stage of the structure for all three strain rates is not changed and is equal to 2 ps.

### 2.3. Potential Function

The simulation model was developed based on the Adaptive Intermolecular Reactive Empirical Bond Order (AIREBO) for the carbon system [45] and the calculations are performed with LAMMPS [46]. As described in Refs. [9,47,48,49,50], the potential function was successfully used to simulate the mechanical and adsorption properties of bulk carbon structures. In Ref. [51] it was shown that a weak ionic bonding is formed between the carbon nanopolymorphs and metal atoms, resulting in a change of the charge at the contact region which can affect contact resistance. Despite this being very important for the understanding of bonding between metal and carbon, it is more often used for the study of graphene growth on a metal substrate, for calculations of binding energies, etc. [51,52,53]. In the present work, peculiarities of interaction between graphene and metal are embedded into potential function [54,55]. Thus, charges and electronic structures in the composite are not considered.

The functions that describe the interactions between Ni and C atoms are referred to the Morse potential with the parameters $D_{e}$ = 0.433 eV, $R_{e}$ = 2.316 Å, β = 3.244 1/Å proposed from ab initio simulation for the Ni–C system [54,55]. The interactions among Ni atoms are also fitted using the Morse potentials with parameters $D_{e}$ = 0.4205 eV, $R_{e}$ = 2.78 Å and β = 1.4199 1/Å obtained in [56]. The potential used in the present work has been successfully applied in the literature to describe the metal-carbide systems [9,40,41,54]. Here, only the physical interaction between Ni nanoparticles and graphene flakes is considered, without considering the possible chemical bonding between them, which is beyond the scope of this paper. Previously it was shown that considering the systems with no-bonding and no-charges, the interactions between the carbon structure and metal surface are mainly attributed to the no-bond van der Waals (vdW) interactions and can be described by Lennard–Jones or Morse potentials [57,58].

MD simulations are conducted using the isothermal-isobaric (NPT) ensemble via a Nose–Hoover thermostat. The equations of motion were solved using a velocity-Verlet algorithm with a time step of 1.0 fs. Periodic boundary conditions are applied along all three directions. During uniaxial tension the volume of the simulation cell remains constant, i.e., as the length of the composite increases in the one direction, its size is correspondingly and anisotropically decreased in the other two directions to maintain the total volume of the system.

An increase in the exposure time during the creation of the composite (from 20 ps to 200 ps) did not change the obtained results. Stress–strain curves are obtained by carrying out at least two modeling processes for each composite’s deformation mode and structural state. The simulation time for dynamic tension at a strain rate $\dot{\varepsilon }=5\times 10^{-3}$ ps${}^{-1}$ at 0 K is 233 ps, while at 300 K the simulation time is slightly longer—236 ps. For a strain rate $\dot{\varepsilon }=5\times 10^{-4}$ ps${}^{-1}$, the simulation time is much longer—1886 ps. Incremental tension at 0 and 300 K at a strain rate $\dot{\varepsilon }=5\times 10^{-3}$ ps${}^{-1}$ requires 264 and 208 ps, respectively. For this regime, with a slower rate $\dot{\varepsilon }=5\times 10^{-4}$ ps${}^{-1}$ at 0 K, 1916 ps is required. It should be noted that the time of the “relaxation” stage of the structure for all strain rates is not changed and is equal to 2 ps for each relaxation step.

The snapshots of the MD results are processed by the Visual Molecular Dynamics package [59]. This is a molecular visualization program that allows you to animate and analyze structural changes obtained by molecular dynamics modeling. With the help of this program, a qualitative analysis is carried out to assess the breaking of bonds and the formation of new ones during uniaxial tension.

## 3. Results

### 3.1. Effect of the Strain Rate

First, the effect of the tension strain rate on the mechanical properties of the fabricated Ni/graphene composite is considered. The system temperature for these simulations is maintained at 0 K. Before uniaxial tension is applied, the structure is relaxed to minimize internal stresses.

#### 3.1.1. Dynamic Load

**Figure 3:**
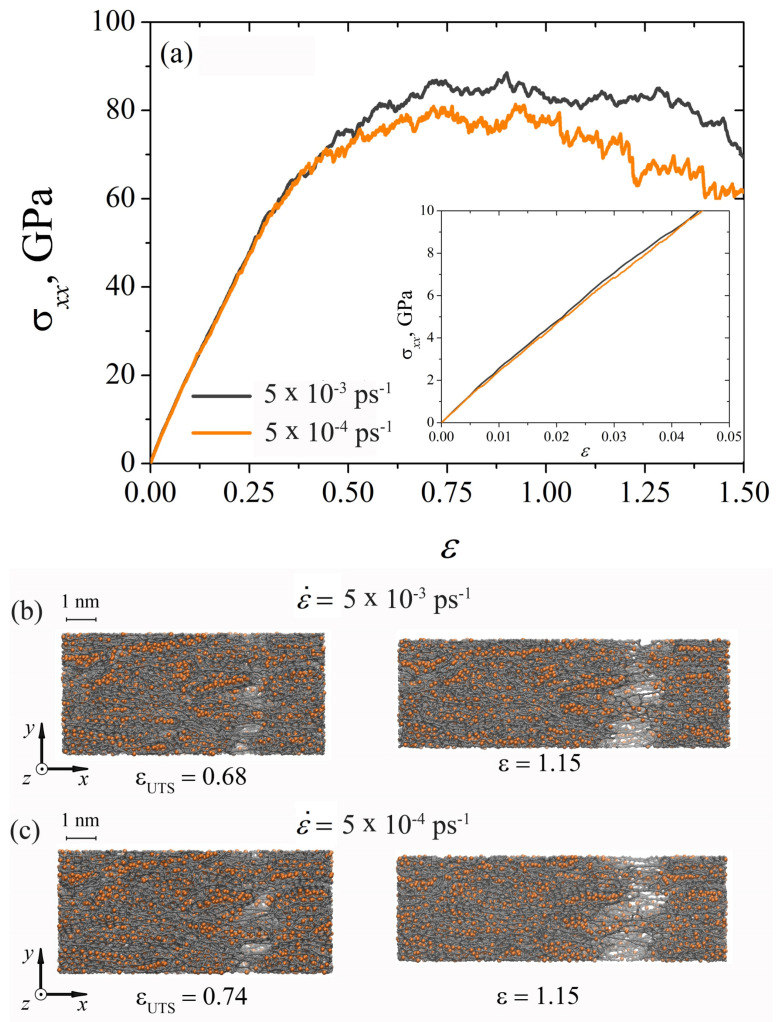
(**a**) Stress–strain curves during uniaxial dynamic tension for Ni/graphene composite at two stress rates: $\dot{\varepsilon }=5\times 10^{-3}$ ps${}^{-1}$ and $\dot{\varepsilon }=5\times 10^{-4}$ ps${}^{-1}$. (**b**,**c**) Snapshots of Ni/graphene composite. Colors as in Figure 1.

Figure 3a shows the stress–strain curves obtained during dynamic uniaxial tension of the composite at two strain rates: $\dot{\varepsilon }=5\times 10^{-3}$ ps${}^{-1}$ and $\dot{\varepsilon }=5\times 10^{-4}$ ps${}^{-1}$. It can be seen that for the elastic regime (see the inset in Figure 3a) there is a complete coincidence of the $\sigma_{xx}(\varepsilon$) curves, thus Young’s modulus is the same for the studied strain rates (see Table 1). For ε>0.25, the curves diverge, and the higher the strain rate, the greater the obtained stress. An abrupt change in the stress state of the composite during dynamic tension is associated with the breaking of carbon bonds and the formation of new bonds. This process is accompanied by a continuous decrease in the stress and is reflected in the drops of the stress on the $\sigma_{xx}(\varepsilon$) curves. The ultimate tensile strength at a strain rate of $\dot{\varepsilon }=5\times 10^{-3}$ ps${}^{-1}$ is higher (see Table 1), but the onset of failure of the composite occurs earlier (at $\varepsilon_{UTS}=0.68$, see Figure 3b). For slower tension of the samples, the rupture of the composite is observed at $\varepsilon_{UTS}$ = 0.74 (see Figure 3c). At that stage for ε>1.15, the rate of dynamic loading does not significantly affect the type of fracture of the Ni/graphene composite (see Figure 3b,c). However, the composite fails faster at a slow strain rate of $\dot{\varepsilon }=5\times 10^{-4}$ ps${}^{-1}$.

#### 3.1.2. Incremental Load

**Figure 4:**
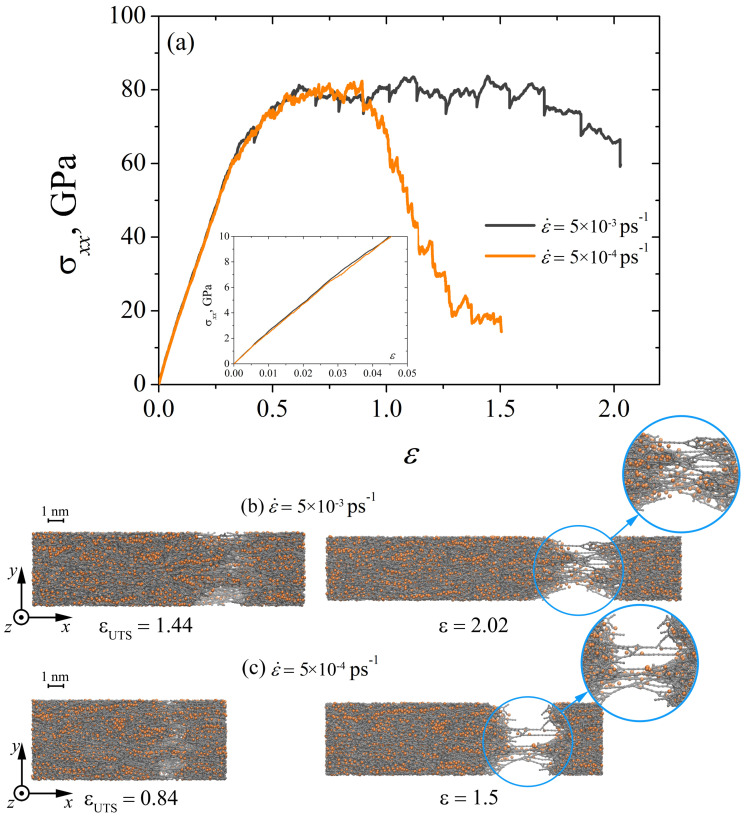
(**a**) Stress–strain curves during uniaxial incremental tension for Ni/graphene composite at different strain rates: $\dot{\varepsilon }=5\times 10^{-3}$ ps${}^{-1}$ and $\dot{\varepsilon }=5\times 10^{-4}$ ps${}^{-1}$. (**b**,**c**) The snapshots of Ni/graphene composite at strain rates 5 $\times \;10^{-3}$ ps${}^{-1}$ and 5 $\times \;10^{-4}$ ps${}^{-1}$, respectively. Colors as in Figure 1.

IIn Figure 4a, the strain-stress curves obtained in the process of incremental uniaxial tension at different strain rates are shown. It can be seen that a decrease in the strain rate $\dot{\varepsilon }$ leads to a significant difference in the deformation behavior of the composite and in the value of ultimate strength $\varepsilon_{UTS}$ (see Table 1). The elongation of Ni/graphene composites during uniaxial tension proceeds due to the formation of long carbon chains. In [39,60,61], the long carbon chains were formed by breaking old carbon bonds and forming new ones. These carbon chains, under incremental uniaxial tension at $\dot{\varepsilon }=5\times 10^{-4}$ ps${}^{-1}$, break fairly quickly at ε> 0.84. Although at $\dot{\varepsilon }=5\times 10^{-3}$ ps${}^{-1}$, the formed carbon chains do not immediately break but elongate as a result of the reconstruction of carbon bonds in the hexagonal graphene structure (see Figure 4b,c). The nickel particles are deformed together with the crumpled graphene flakes. Young’s modulus under incremental loading does not depend on the strain rate of the composite, which is also typical for dynamic uniaxial tension.

### 3.2. Effect of Temperature

#### 3.2.1. Dynamic Load

The effect of temperature on the mechanical properties of Ni/graphene composite is studied for both methods of uniaxial tension at a strain rate of $\dot{\varepsilon }=5\times 10^{-3}$ ps${}^{-1}$.

**Figure 5:**
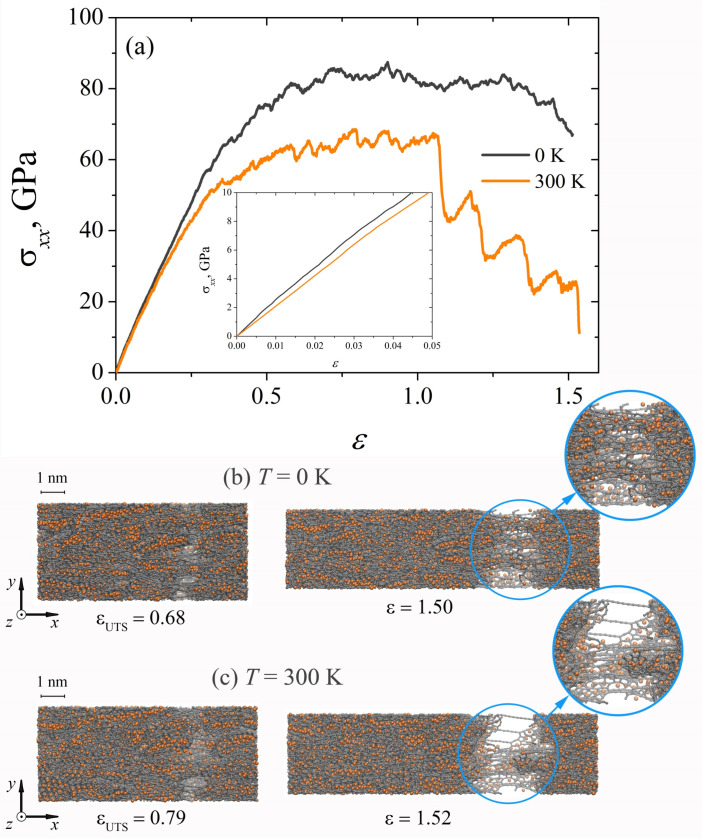
(**a**) Stress–strain curves during uniaxial dynamic tension at $\dot{\varepsilon }=5\times 10^{-3}$ ps${}^{-1}$ for two temperatures: 0 K and 300 K. (**b**,**c**) The snapshots of Ni/graphene composite at 0 and 300 K, respectively. Colors as in Figure 1.

In Figure 5a, the stress–strain curves obtained by dynamic uniaxial tension of a Ni/graphene composite at 0 and 300 K are presented. It can be seen that at zero temperature the ultimate tensile strength of the composite is much higher than at 300 K. Up to the strain ε=1.0, the course of the $\sigma_{xx}(\varepsilon$) curves does not depend on the temperature (see Figure 5a), while after ε>1.0 the decrease of stresses can be observed at 300 K. Thus, as a result of the breaking of the carbon bonds, long carbon chains begin to form (see Figure 5c at ε=1.52). Subsequently, a large number of these chains are broken, which leads to a sharp decrease in stress. However, to replace the destroyed carbon chains, new ones are formed again according to the same scheme, which are subsequently destroyed, again leading to another drop in $\sigma_{xx}$. This process is repeated over and over again until the composite is completely destroyed.

This deformation mechanism is not observed at 0 K, since thermal fluctuations of atoms play a key role in the formation of long carbon chains. At 300 K, the hexagonal graphene lattice is easily transformed to form long covalent carbon chains. Carbon chains are also formed during low-temperature tension of the composite, but chains are shorter than at 300 K (see Figure 5b,c), and after their rupture, new chains are practically not formed.

#### 3.2.2. Incremental Load

**Figure 6:**
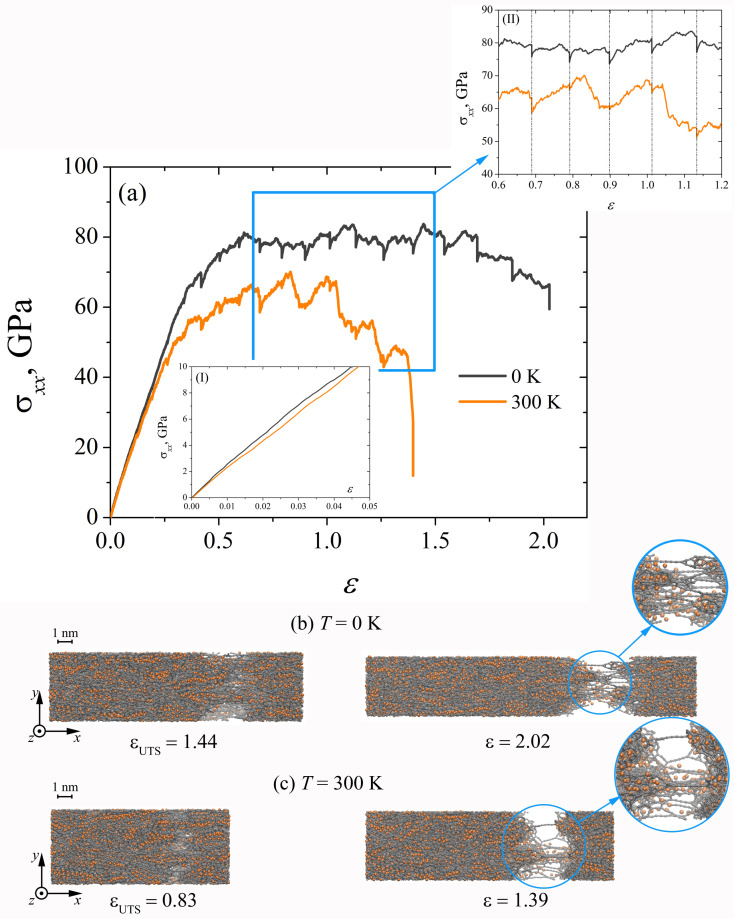
(**a**) Stress–strain curves during uniaxial incremental tension at $\dot{\varepsilon }=5\times 10^{-3}$ ps${}^{-1}$ for two temperatures: 0 K and 300 K. Inset (I) shows the elastic regime and inset (II) shows the difference of the $\sigma_{xx}$ at 0.6<ε<1.2. The dashed-dotted lines in inset (II) corresponds with the “relaxation” stages between uniaxial tension stages. (**b**,**c**) The snapshots of Ni/graphene composite at 0 and 300 K, respectively. Colors as in Figure 1.

Figure 6a shows the stress–strain curves obtained as a result of uniaxial incremental tension at 0 and 300 K. It can be seen that in the region of elastic deformation (inset I in Figure 6a) the $\sigma_{xx}(\varepsilon$) curves do not coincide. This indicates different values of Young’s modulus. Thus, Young’s modulus decreases with a temperature increase, which is typical for most metallic and carbon materials [62,63,64]. Note that the same change in Young’s modulus is also typical for dynamic uniaxial tension (see the inset of Figure 5a).

The ultimate tensile stress and strain of the composite decreased with temperature increase, which is associated with a decrease in the strength of interatomic interaction due to thermal fluctuations of atoms. The resulting carbon chains under uniaxial tension (Figure 6b,c) are destroyed rapidly at 300 K. Inset (II) of Figure 6a shows that the “relaxation” stage (dotted lines) is accompanied by a reduction in stress due to structure reconstruction (breaking of carbon bonds and the formation of new ones). Note that, first of all, those carbon bonds that are oriented perpendicular to the tension direction are broken. After the end of the “relaxation” stage, $\sigma_{xx}$ slightly increases again. At 0 K, a significant stress drop is observed only at the “relaxation” stage. However, at 300 K, sigma decreases both at the “relaxation” stage and during uniaxial tension, for example, at 0.83<ε<0.9 or 1.04<ε<1.13. This is due to the multiple breakages of previously formed carbon chains, which can no longer elongate (see Figure 6b,c).

### 3.3. Uniaxial Tension during Incremental Load

The material properties can considerably depend on the direction in which these properties are measured, which is anisotropy. As it was shown for Ni/graphene composite, fabrication of the composite with a more homogeneous structure enhanced mechanical properties [22]. The strength properties of Ni/graphene composite strongly depend on the direction of the uniaxial tension under dynamic regime [39]. Thus, changes in the strength of the composite during incremental deformation along different tension axes should also be considered. The strain rate is $\dot{\varepsilon }=5\times 10^{-4}$ ps${}^{-1}$ and the test temperature is 0 K.

**Figure 7:**
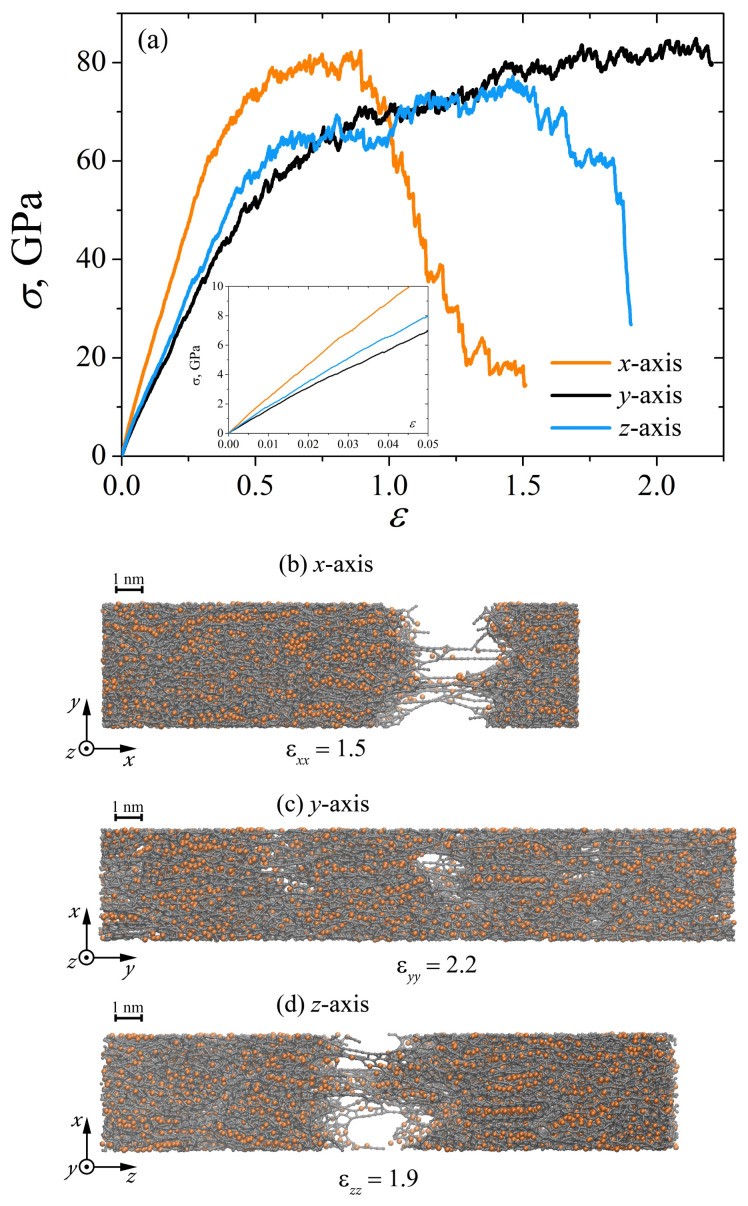
(**a**) Stress–strain curves during uniaxial incremental tension along the *x*, *y* and *z*-axes at strain rate $\dot{\varepsilon }=5\times 10^{-4}$ ps${}^{-1}$. (**b**–**d**) The snapshots of Ni/graphene composite during incremental deformation along the *x*, *y* and *z*-axes, respectively. Colors as in Figure 1.

Figure 7a shows that the mechanical properties of the Ni/graphene composite strongly depend on the tension direction under incremental regime. The composite has the highest tensile strength under tension along the *x*-axis, however, plasticity under these deformation conditions is the lowest, while when stretched along the *y*-axis, the composite has the maximum plasticity. At the same time, at the strain $\varepsilon_{yy}=2.2$ fracture of the composite just starts, which can be observed from the snapshot of the structure [Figure 7c], where a small number of pores formed in the central part of the composite. Note that when the structure is under tension along *x* and *z*-axes, the deformation occurs as a result of the formation of long carbon chains with their subsequent fracture. Such a difference in the strength of the composite is primarily associated with the initial structure of the Ni/graphene composite (see Figure 1, structure A). The proposed high-temperature treatment is aimed to obtain a composite with low anisotropy and improved mechanical properties. Despite this simulation technique leading to the formation of a more anisotropic structure, it is not enough to create a composite with isotropic properties in all directions. Note that, at a lower strain rate ($\dot{\varepsilon }=5\times 10^{-3}$ ps${}^{-1}$), the mechanical properties of the Ni/graphene composite are also affected by the direction of the uniaxial tension.

## 4. Discussion

**Figure 8:**
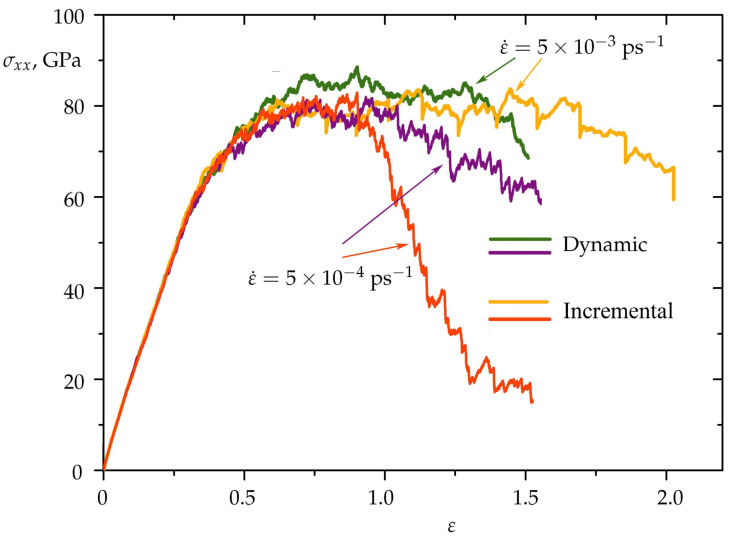
Stress–strain curves during uniaxial dynamic (green and violet curves) and incremental (orange and red curves) tension along *x*-axis for strain rate $\dot{\varepsilon }=5\times 10^{-3}$ ps${}^{-1}$ and $\dot{\varepsilon }=5\times 10^{-4}$ ps${}^{-1}$ at 0 K.

For correlation analysis of dynamic and incremental uniaxial tension at different strain rates, Figure 8 shows the stress–strain curves obtained at 0 K. It can be seen that the tension rate affects the plasticity of the Ni/graphene composite under incremental deformation. The higher the strain rate, the greater the value of strain until complete fracture.

At a strain rate of $\dot{\varepsilon }=5\times 10^{-3}$ ps${}^{-1}$ (Figure 8), the tensile strength of the Ni/graphene composite under dynamic load is higher than under incremental load. It can be assumed that the reason is the distribution of stresses in the composite at the “relaxation” stage, which leads to a certain decrease in the critical tensile stress. At a strain rate, $\dot{\varepsilon }=5\times 10^{-4}$ ps${}^{-1}$, a difference in tensile strength is not observed, however, the formation of long carbon chains occurs faster under incremental uniaxial tension, and therefore the composite is broken faster. Although these two manners of strain application result in a similar scenario, values of the fracture strength of covalent systems for dynamic loading can be overestimated. As it is mentioned in [44], under dynamic load the covalent networks exhibit brittle fracture instead of ductile because of insufficient structure relaxation.

**Figure 9:**
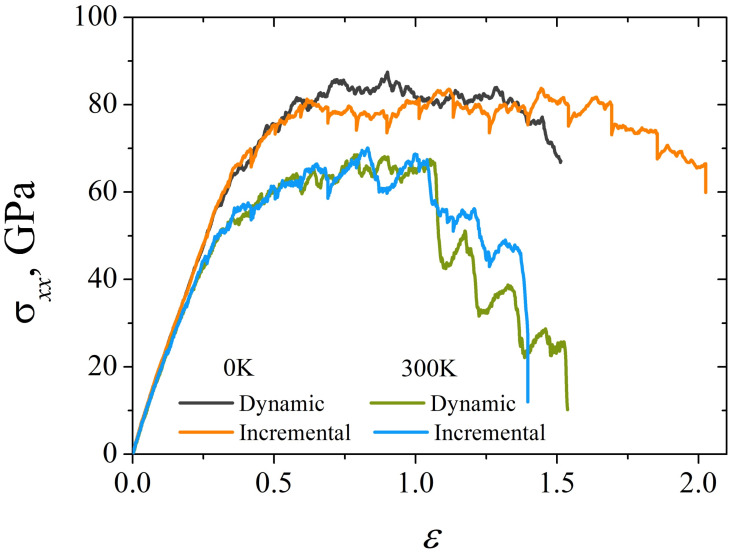
Stress–strain curves during uniaxial tension for dynamic (black and green curves) and incremental (orange and blue curves) deformation at 0 and 300 K. Strain rate is $\dot{\varepsilon }=5\times 10^{-3}$ ps${}^{-1}$.

The temperature significantly affects the tensile strength of the Ni/graphene composite, which can be seen in Figure 9. At 300 K, the strength of the composite is 17–23% lower than at 0 K. Such a difference in $\sigma_{xx}$ is associated with thermal fluctuations of atoms in the lattice, which weaken the interatomic bonds and, consequently, reduce the composite strength. However, the uniaxial tension methodology practically does not cause a change in the stress–strain state of the composite. Figure 9 shows that the stress-strain curves for dynamic and incremental uniaxial tension almost completely coincide at the same temperature. Note that at 0 K, dynamic tension leads to faster fracture of the Ni/graphene composite than under incremental load. This is due to the redistribution of stresses at the stage of “relaxation” of the structure under uniaxial incremental tension.

Young’s modulus (*E*) of the obtained composite are 219 GPa (at 0 K and $\dot{\varepsilon }=5\times 10^{-3}$ ps${}^{-1}$), 313 GPa (at 300 K and $\dot{\varepsilon }=5\times 10^{-3}$ ps${}^{-1}$) and 218 GPa (at 0 K and $\dot{\varepsilon }=5\times 10^{-4}$ ps${}^{-1}$). For comparison, Young’s modulus of the Ni-graphene composite obtained by electrodeposition is 240 GPa [12], which is in a good agreement with the obtained results.

**Figure 10:**
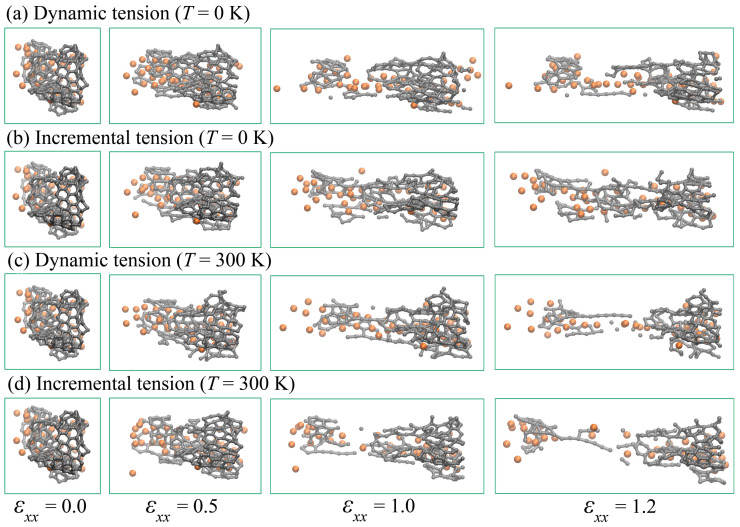
Snapshots of a single structural element of a Ni/graphene composite under dynamic (**a**,**c**) and incremental (**b**,**d**) uniaxial tension at 0 (**a**,**b**) and 300 K (**c**,**d**). Strain rate is $\dot{\varepsilon }=5\times 10^{-3}$ ps${}^{-1}$. Colors as in Figure 1.

Figure 10 shows the snapshots of a GF located in the center of the Ni/graphene composite at different strains $\varepsilon_{xx}$ during tension at 0 and 300 K. As it can be seen, nanoparticles completely lose their initial round shape, and separated Ni atoms spread over the graphene flake. Thus, after such strong compression, this is no longer a nanoparticle, but a graphene network with metal atoms on its surface. The interaction energy between Ni and graphene is strong [52], as Ni atoms are attracted to the flake, which results in the destruction of the nanoparticle. At each strain rate, metal atoms move together with GF; however, mechanical behavior depends on the tension strain rate. It can be seen that, regardless of the deformation type at room temperature (Figure 10c,e), the graphene flake, together with nickel particles, is broken faster than at 0 K (see Figure 10b,c). This is due to thermal fluctuations of atoms, which facilitate the breaking of old carbon and nickel bonds, leading to the formation of long carbon chains during further tension. Analysis of the effect of strain rate on the behavior of a single structural element at different strain rates showed that at a lower strain rate $\dot{\varepsilon }=5\times 10^{-4}$ ps${}^{-1}$, faster destruction of GF occurred. As a result, the amount of deformation before the destruction of the composite is reduced. However, the type of tension (incremental or dynamic) does not have a significant effect on the process of fracture of a single element of the composite.

**Figure 11:**
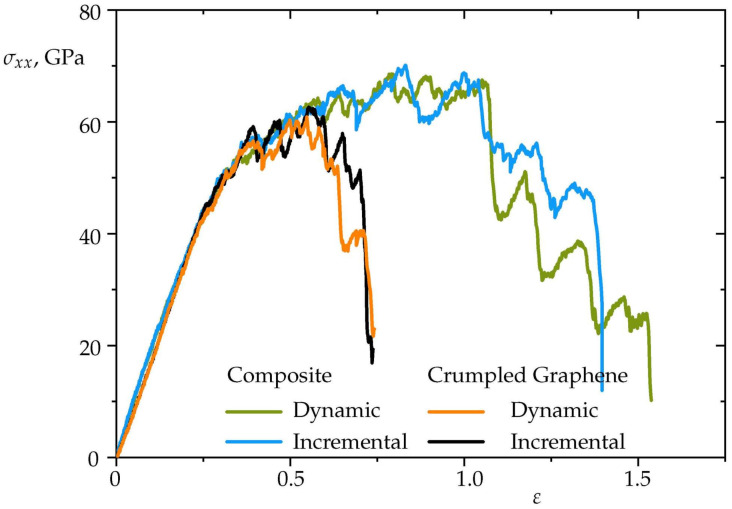
Stress–strain curves during uniaxial tension for incremental (black and blue curves) and dynamic (orange and green curves) tensile loading at 300 K. Strain rate is $\dot{\varepsilon }=5\times 10^{-3}$ ps${}^{-1}$.

In Figure 11, stress–strain curves during uniaxial tension of two types (dynamic and incremental) at 300 K for Ni/graphene composite and for pure crumpled graphene are presented for comparison. The ultimate tensile strength of the crumpled graphene at given conditions is about 60 GPa for both dynamic and incremental loading. As well for composite, curves for dynamic and incremental loading are very close in elastic and plastic regimes. For Ni/graphene composite, $\sigma_{UTS}$ is about 65 GPa. However, critical strain values are very different for composite and CG, ε = 1.0 and ε = 0.6, respectively. This shows that metal nanoparticles improve the ductile behavior of crumpled graphene.

From this point of view, a comparison with the tensile behavior for a pure metallic sample should also be added. However, from our previous studies, it was concluded that such a comparison is not reasonable. In [9,65], where such comparison is conducted, especially metal matrix composites are considered: graphene network is the reinforcement for the metallic sample under sintering. For such composites, where there are more metallic atoms than carbon atoms, it can be clearly seen that graphene increases the strength of the metal matrix. In the present work, graphene network is the matrix for the small number of metal nanoparticles. From this point of view, a comparison with crumpled graphene is much more reasonable.

Earlier, similar composite structures were studied in [9,22,66]. The carbon matrix can be considered as the network integrated into the Ni matrix as an interlocking structure. The structure obtained in the present work is consistent with the experimental observations. The strength of such a composite is explained by the covalent bonds that appeared on the edges of the flakes and provide connections among GFs [9,50,66,67]. The strength of the obtained composite is much larger than for convenient metal matrix composites reinforced by graphene layers [68], where the ultimate tensile strength of the Ni matrix with a single graphene layer is about 14 GPa. In common, the strength of such composites increases due to the presence of dislocations that interact with the graphene plane. The high strength of composites with graphene networks showed better dislocation-blocking than for composites with graphene sheets. Here, the obtained composites are much stronger because of the covalent graphene network, while Ni atoms increase the ductility. For similar composites based on a graphene network, the ultimate tensile strength is found to be 40 GPa [65]. However, the strength is two times less than for the composite obtained in our work since the metallic part of the composite from [65] is much bigger, which weakens the structure. The same knit-like mechanisms of fracture were shown for graphene at different conditions and is reviewed in Ref. [69].

This study illuminates the relationship between the simulation technique and mechanical properties of the metal/graphene composite, conducing the development and comprehensive utilization of such structures. Both studied methods of tension (dynamic and incremental) give close results on ultimate tensile strength and Young’s modulus. The formation of long carbon chains occurs faster under incremental uniaxial tension, and therefore the composite is broken faster. It can be concluded that the chosen strain rate should be reasonably small to obtain physically correct results, but not too small, which enlarges the simulation time. For such composite material, a strain rate of $\dot{\varepsilon }=5\times 10^{-4}$ ps${}^{-1}$ and lower can be recommended.

## 5. Conclusions

A simulation approach to study Ni/graphene composite under uniaxial tension at different simulation conditions by means of MD has been presented. Incremental and dynamic tension techniques, strain rate, and simulation temperature are chosen as the key factors in order to obtain a satisfactory simulation methodology for the simulation of metal/graphene composites. It is shown that a decrease in strain rate, as well as simulation at room temperature for such structures, can give lower ultimate tensile strength under uniaxial tension since it allows for slower structural transformations. However, dynamic and incremental mechanisms of tension lead to similar mechanical behavior, though the incremental regime of the simulation is much longer.

Overall, the strengthening of the composite with a graphene network composed of graphene flakes is shown. The route for composite fabrication plays a key role in the formation of the composite with low anisotropy. The considerable difference in the composite strength under uniaxial tension along three directions is shown. However, in [65] it was shown that for the same type of a composite with graphene network the tensile responses in three directions show similar trends despite small differences in values caused by the small distinctions of atomic configurations. The ultimate tensile strength of the Ni/graphene composite is close to that of a pure graphene network, while the ductility of crumpled graphene with metal nanoparticles inside is two times higher.

## Figures and Tables

**Table 1:** Numerical characteristics of the mechanical properties of Ni/graphene composites: ultimate tensile strength $\sigma_{UTS}$ and ultimate tensile strain $\varepsilon_{UTS}$, Young’s modulus *E*.

	Dynamic Tension			Incremental Tension		
$\dot{\varepsilon }=5\times 10^{-3}$ ps${}^{-1}$						
*T*, K	*E*, GPa	$\sigma_{UTS}$, GPa	$\varepsilon_{UTS}$, GPa	*E*, GPa	$\sigma_{UTS}$, GPa	$\varepsilon_{UTS}$, GPa
0	219	88	0.91	219	83	0.83
300	313	68	0.79	313	69	0.83
$\dot{\varepsilon }=5\times 10^{-4}$ ps${}^{-1}$						
0	218	81	0.92	218	82	0.89

## Data Availability

Data available on request due to privacy/ethical restrictions.

## Abbreviations

The following abbreviations are used in this manuscript:

MD	Molecular Dynamics
CG	Crumpled Graphene
GF	Graphene Flake
